# The Boarding out of Pauper Children

**Published:** 1889-09-21

**Authors:** 


### The Boarding Out of Pauper Children.

II.—MISS MASON'S REPORT.

In a former article some account was given of the system of boarding out pauper children, attention was called to the special dangers attending it, and we printed in some detail the Orders issued by the Local Government Board, in the present year, for the guidance of guardians, voluntary committees and visitors, foster-parents, medical officers, and schoolmasters.

To-day we turn to the comprehensive report of Miss M. H. Mason, the inspector appointed by the Board in connection with this special and important section of poor law administration. The children found still chargeable to the rates numbered 326, and were boarded out under twenty-one committees in the counties of Cambridge, Chester, Cumberland, Essex, Hants, Norfolk, Surrey, Suffolk, and Westmoreland. They are nearly all London children. Some few are sent to committees from the unions of Carlisle, West Derby, Croydon, Eastbourne, and Horsham, but the centres from which the great bulk of the children are sent bear the familiar names of Whitechapel, St. Pancras, Holborn, Kensington, Bethnal Green, Lambeth, Paddington, St. Marylebone, Poplar, Wandsworth, and Clapham.

Whitechapel and Windermere are some space apart even in actual distance, but still wider asunder in our thought and association of ideas. As one studies the boarding-out system, a few moments are demanded for a discovery like this—Whitechapel children brought up at Windermere! This is, unquestionably, a great and beneficial change for "orphan and deserted" pauper children, but the crowning value of the system seems to be that the change is for life. They are taken away, once for all, not only from the associations of Whitechapel, but from the associations of the workhouse. The children are not merely relieved, but taken up out of the ranks of pauperdom, given a place and a start in life, a real opportunity of joining the wage-earning, industrious, and respected classes of the community.

What kind of people constitute the committees who find homes and foster-parents for these children, and co-operate with the guardians in all that concerns their welfare? People who are themselves in easy circumstances, and wish to make life as bright and hopeful as possible for those who are sadly handicapped in the race—sinned against, not sinning; people who here and there have crotchets of their own, who will sometimes be blind to an abuse rather than offend some local cottager, but who are in the main already doing their voluntary work right well, and are full of anxiety to learn how to do it better. Miss Presseur, of Windermere, for example, has been much identified with the boarding-out system, and is president of the local committee responsible for thirty-nine girls from Bethnal Green, Paddington, Poplar, St. Pancras, and Whitechapel. "The entire clothing of almost all these children is given by Miss Presseur herself, and the rest by other members of the committee, no allowance being made for it by the guardians. It was far better in quality and greater in quantity (Miss Mason tells us) than absolutely necessary, every possible want being supplied; but it was plain, and quite suitable for their station in life. Every Saturday afternoon the children are invited to Miss Presseur's house, and play in her garden in the summer. Every year she gives them and their foster-parents a garden-party, but this year, not being well enough to entertain them, she hired a steamer, and sent them for a picnic across the lake. If needing it they are sent to the sea-side, or on visits elsewhere, for change of air. Three were thus sent while I was at Windermere. If any girl displays a taste for some special pursuit, it is cultivated. Miss Presseur was herself giving drawing lessons to one girl while I was there."

This is, of course, an exceptional case, as the reader would be more fully persuaded if details were given of many other acts of kindness towards the children whilst still chargeable to the rates and after they have ceased to be chargeable. But, although exceptional, there is much in the instance we have taken which is typical of the work done, and the interest taken in the children by these voluntary committees. The fact is, ladies and gentlemen who feel pity for the undeserved distress of these helpless orphan and distressed children, are asked to throw this pity into active benevolence by helping in the care and training of them. Responding to the call, their work is a labour of love, and this affectionate guardianship, in whatever part of the country it is undertaken, will be ingenious in discovering means to promote the happiness and welfare of the children. The boarding-out system multiplies tenfold the guardians of the poor, and attracts the most suitable and efficient to a work which really belongs to all.

Her Royal Highness the Duchess of Albany presides over the Esher Committee. Here, "The children's clothing was made by the ladies themselves, and the fact that the stockings were knitted by them, and that they might come to try them on at any time, made the foster-parents careful to keep the children's feet always well washed. Rents being very high here, no foster-parents could be found to take a child for less than 6s. weekly maintenance, besides clothing, school fees, and doctors; and the committee were themselves paying an additional 2s. weekly for each child." The secretary of the Rowhams Committee (Hants) has set up a convalescent home in the village, where the children are sent if unwell, and if there is anything more serious the matter with them, they are sent to a children's hospital about two miles off. In many cases, those acting on committees have the means as well as the inclination to provide everything that is really needful for the children; but we believe that all so engaged have, without exception, what is far more essential than extras and luxuries, namely, real affection for the little waifs and strays whose future they are shaping by present kindness and attention.

Miss Mason mingles the grave and gay in her references to the cleanliness and health of the children. She asked one girl when her feet were washed. "Mother says the time's not come yet." "When will the time come?" Miss Mason asked. "In the summer," replied the child. It was then the middle of March. In another case, a foster-mother explained that a girl was now too old to wash more than her face and neck, and that a skin complaint which Miss Mason had discovered "came below the water-mark." We are assured that instances of this kind are so common, that no special notice is taken of them unless they are found associated with more serious neglect of ordinary cleanliness and comfort. We have known good judges condemn a writer of fiction of utter ignorance of human nature, because the hero was a man who kept himself faultlessly clean and his house abominably dirty; but Miss Mason tells us she has very commonly found going together a dirty house and clean children, or clean children and a dirty house. It is curious, at least. Even where foster-parents are people of one idea—if that one idea be cleanliness, it ought to be large enough to include both the house and its occupants.

Will our readers share Miss Mason's toleration on the question of "lice in the head"? Allowing that if thoroughly neglected they will eat sores into the face and neck, yet these cases are reported only when *pediculi capitis* are allowed to increase to an unpardonable extent. The pardonable extent is not defined. This is Miss Mason's position: "The mixing of the children's hats and clothes at school, where they are crowded on the same pegs, makes it impossible for the cleanest of mothers to keep their children's heads always free from them, or their eggs, commonly called 'nits.' Body-lice (*pediculi corporis*) are, however, quite inexcusable." We are disposed to urge a stricter rule, and Miss Mason herself gives a good instance of how much care and attention can effect. In a surprise visit, she tells us, "I found two boys evidently neglected, and literally covered with flea-bites, their linen being marked all over. Their foster-parents cleaned them in consequence of my discovery, and when I re-examined them a week afterwards there was not a trace of the fleas left." Should the *pediculi capitis* be allowed more quarter?

But there was one series of cases in which the health of the children was directly involved as well as their cleanliness. The "Denmead incident" has caused a considerable stir, and it has been the wisdom of some local Solomons to complain loudly that the inspector had several poor children effectually cured of "itch." If this matter were one of mere controversy it would be easy to deal out blame to the foster-parents, the committee, and even the doctors. Miss Mason takes the more rational ground that all human beings are fallible, and that ignorance of the matter in question, or misunderstanding of the course to be pursued under certain circumstances, will account for all that happened. When we remember that doctors, foster-parents, and committee gave every facility in having matters set right, we shall be the more willing to endorse Miss Mason's reasonable view of the whole matter. Thorough examination of the children, as opposed to mere superficial inspection—this is the important and permanent lesson from what has happened at Denmead (Hants). The inspector on undoing the collar of one boy and looking down his neck, found that his neck was covered with a rash. This led to more complete examination, and this boy and some of his companions were found suffering from itch and *pediculi corporis*. It was a home in which too many children were lodged, just one of these cases provided against in the new Orders of the Local Government Board. "Itch" seems to have been rife in the district. In other homes the children were found suffering from it, and a case is mentioned of the doctor attending some of these children, possibly owing to some misunderstanding, not communicating the nature of the complaint either to the committee or the foster-parents.

We may rest certain that children boarded out through voluntary charity, as well as the children chargeable to the rates, will be more thoroughly examined henceforward, and we are indebted to Miss Mason for bringing about this needed reform. She has not forgotten the practical advice given her by the dying woman in one of her father's cottages—advice which is now recognised on the largest scale: "Do not be taken in, as ladies so often are, with outside appearances, but take off the poor little things' boots and stockings and see whether their feet have been properly attended to, and turn up the girls' petticoats to see what they have got on underneath."

